# Contralateral patent processus vaginalis repair in boys: a single-center retrospective study

**DOI:** 10.1038/s41598-022-15435-9

**Published:** 2022-07-15

**Authors:** Liu Jinxiang, Cao Qingwei, Qiu Shenghua, Xia Yunqiang, Liu Haiyang, Liu Chengliang, Xu Meng

**Affiliations:** 1 Master of Medicine, Linyi Central Hospital, Linyi, Shandong China; 2 Bachelor of Science in Medicine, Linyi Central Hospital, Linyi, Shandong China

**Keywords:** Paediatric research, Epidemiology

## Abstract

To ascertain the prevalence of contralateral patent processus vaginalis (CPPV) in life and the significance of the prevalence trends for treatment. We performed a retrospective review of all inguinal hernias (IHs) that underwent repair in our hospital from 2014 to 2018. We analyzed the frequency of occurrence and treatment in boys. We assessed and compared the history, initial sides of hernia, CPPV and prognoses in different age groups. We assessed all IH cases repaired in our hospital and selected male patients of a variety of ages, including boys and men. Recurrent cases were not enrolled. A total of 3243 cases were enrolled: 2489 [right-sided IH 1411 (56.69%) vs. left-sided IH 975 (39.17%), bilateral IH 103 (4.14%)] in children and 754 [right-sided IH 485 (64.32%) vs. left-sided IH 236 (31.30%), bilateral IH 33 (4.38%)] in adults. A total of 1124 CPPVs were identified in children with unilateral IH (2386), and 12 were identified in adults (267) (p < 0.0001). There were no significant differences in recurrence rate between different subgroups of children (p > 0.05). The incidence of IH in boys was significantly higher than that in men. The number of incident cases declines rapidly with age in boys. The processus vaginalis is normally obliterated and involuted but may instead remain patent for a long period before closure; routine exploration on the contralateral side may eliminate the possibility of spontaneous PPV closure.

## Introduction

Inguinal hernias (IHs) need to be repaired to prevent them from becoming incarcerated; this is true of both children and adults. With the introduction and promotion of laparoscopic repair in children, a large number of contralateral patent processus vaginalis (CPPV) cases have been exposed and repaired. Whether CPPV needs to be repaired, however, is debatable. Based this study, we infer that CPPV should not be repaired routinely.

During laparoscopic hernia repair, CPPV is sometimes closed simultaneously^1^. A number of studies have shown that the incidence of CPPV is 50–70%^2,3^, and the incidence of IH and CPPV is age dependent^4,5^. In this study, we investigated the characteristics of CPPV over the lifespan.

## Workflow

Almost all incarcerated IHs in children and adults were reduced by gentle manual pressure first if the patients’ state permitted. Routine operation was not recommended for children younger than 6 months^6^ unless there was a strong demand from the parents. Surgical treatment is possible, but a noninvasive strategy can reduce the risk associated with anesthesia, and some children may heal without surgery. The hospital is a regional medical center and a comprehensive Grade 3A hospital. Pediatric Surgery and General Surgery are two separate departments of the hospital. Laparoscopic hernia repair began in children in 2004 and in adults in 2017.

## Patients and methods

### Patients

This study was approved by the Clinical Research Ethics Committee of Linyi Central Hospital. A retrospective study was carried out on all male patients with IH who visited the hospital between 2014 and 2018. Patients under 15 years old were classified in the child group and underwent repair in the pediatric surgery ward.

The rest were in the adult group and underwent repair in the general surgery ward. Recurrent cases were excluded from this study. The medical history and condition of boys were provided by their parents or guardian, and those of adult men were provided by the patients themselves or their close relatives.

### Group comparisons

#### Child group and adult group

**Figure 1:**
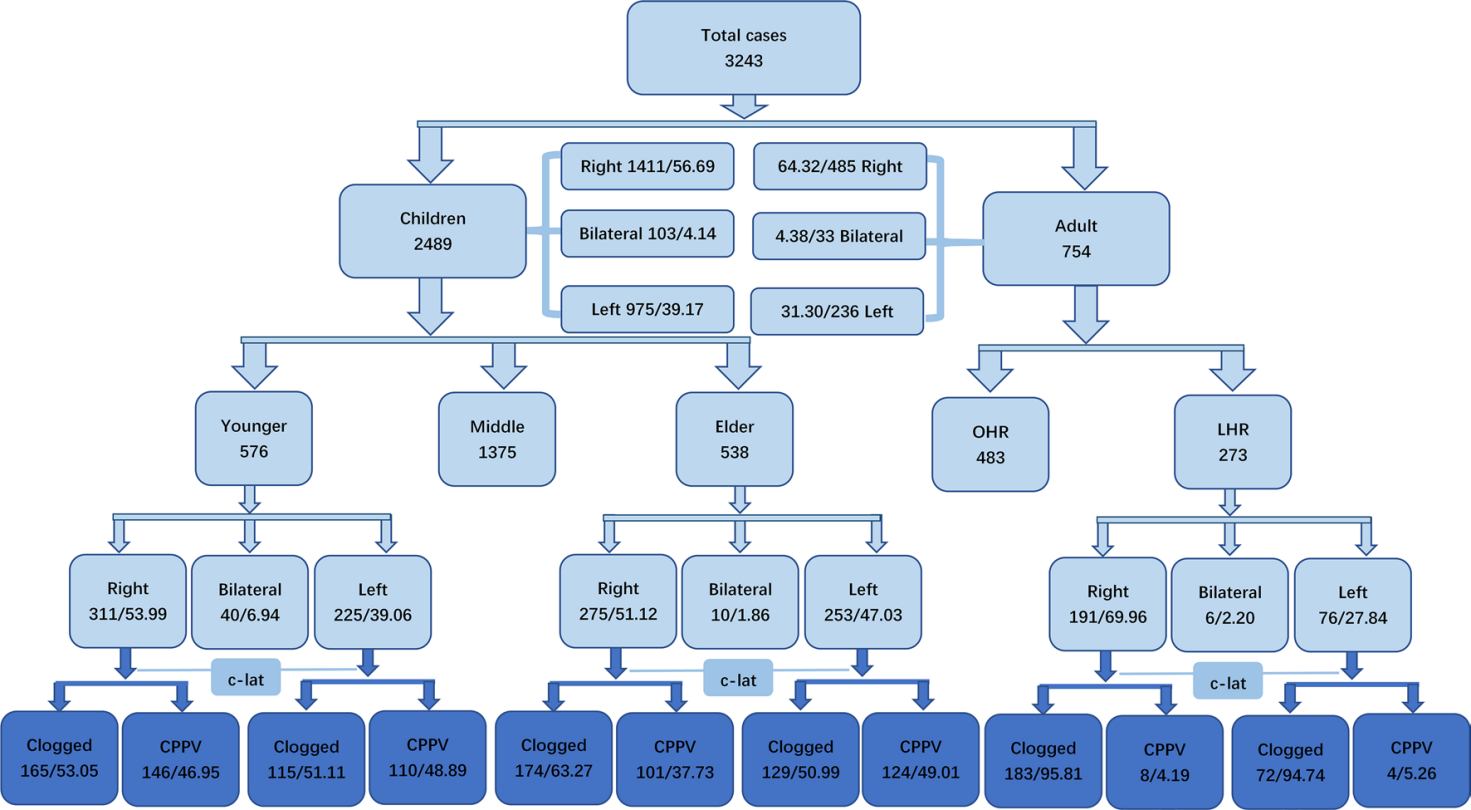
The incidence of CPPV in different groups. Younger: aged ≤ 18 months, older: aged ≥ 60 months, c-lat: contralateral.

The variables evaluated were sex, age at operation, history, initial side of hernia, and development of contralateral hernia (Fig. 1).

#### Younger and older groups of children

Children younger than 18 months and older than 60 months were selected and divided into a younger group and an older group. The variables evaluated were sex, age at operation, history, initial side of hernia, and development of contralateral hernia.

#### Follow-up

All children were followed up for at least 2 years. The forms of follow-up included telephone surveys, WeChat conversations, and outpatient visits. After IH repair, we provided instructions on how to monitor for hernia recurrence; if there were clinical manifestations suggesting recurrence, the patient returned to the outpatient setting as soon as possible. We analyzed the total recurrence rate in boys on all examined sides and on the repaired side, hernia side, CPPV side and clogged side.

### Statistical analysis

Demographic data, such as age, medical history, hernia side, and number of CPPVs detected by laparoscopy, were collected. Continuous data are expressed as the mean (s.d.) and analyzed using a two-sample t test. Χ^2^ tests were used to determine the significance of differences in the incidence of CPPV with regard to side. All tests were two-sided, and p < 0.05 was considered statistically significant.

### Ethics approval

For all cases covered in the submission, signed informed consent was provided by the patients and/or relatives who were legally responsible for them. The signed medical records were stored in the medical records room of Linyi Central Hospital. The clinical diagnosis and treatment were in compliance with the World Medical Association Declaration of Helsinki and the Council for International Organizations of Medical Sciences International Code of Biomedical Ethics Involving Human Beings.

## Results

### Clinical data

From 2014 to 2018, a total of 3243 cases were recorded; the mean age was 14.74 ± 23.14 years, the minimum age was 4 months, and the oldest age was 93 years old. The mean length of medical history was 1.63 ± 5.62 years, the longest was 70 years, and the patient was a 78-year-old man with the appearance of the initial symptoms when he was 8 years old (51 cases with onset ages under 15 years old, accounting for 6.76% of the sample).

**Figure 2:**
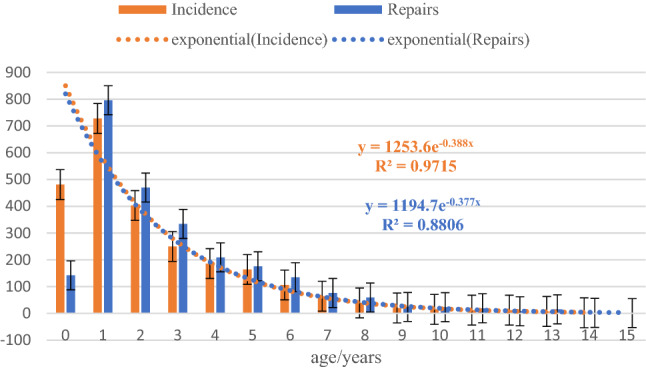
Trend chart of onset and treatment ages in children. Within a few years of birth, both incidence and repairs declined rapidly.

In the pediatric group, the mean age was 3.36 ± 2.47 years, the mean medical history was 0.74 ± 2.71 years, there were 1409 right-sided IHs (56.69%), 975 left-sided IHs (39.17%), and 103 bilateral IHs (4.14%) (Table 1). Statistics showed that both incidence and repairs declined rapidly, beginning at a young age (R^2^ = 0.9715, 0.8806) (Fig. 2).

**Table 1:** Clinical details included in the study.

	Boys		Men		t/χ^2^	p
Total (T)	2489		754			
Overall mean age (years)	14.73 ± 23.14					
Subgroup mean age (years)	3.36 ± 2.47		53.65 ± 17.40			
Min. age (months)	4					
Max. age (years)			93			
Subgroup median age (years)	2.5		55			
Median age (years)	8		54			
Case ratio	3:1					
Age-range ratio	1:5					
Right (R)/%	1411	56.69	485	64.32	15.39	0.0001^#^
Left (L)/%	975	39.17	236	31.30		
Bilateral (B)/%	103	4.14	33	4.38	0.082	0.7747^##^
History (years)	0.74		4.57		17.10	< 0.0001^###^

^#^χ^2^ test, ^##(^R + L) vs. B, χ^2^ test, ^###^unpaired t test.

In the adult group, the mean age was 53.65 ± 17.40 years, and the mean length of medical history was 4.57 ± 10.00 years (children vs. adults, t = 17.10, p < 0.0001). There were 487 right-sided IH cases (64.32%), 236 left-sided IH cases (31.30%) (children vs. adults, χ^2^ = 15.39, p < 0.0001), and 33 bilateral IH cases (4.38%) (χ^2^ = 0.082, p = 0.7747) (Table 1). The case ratio of children to adults was 3:1, and the age-range ratio of the groups was 1:5.

### Children vs. adults

On laparoscopy, 1124 (47.11%) CPPVs were noted in 2386 unilateral IHs of children, and only 12 (4.49%) %) CPPVs were checked in 267 unilateral IHs of adults (χ^2^ = 178.1, p < 0.0001). CPPV on the right side was identified in 50.77% of the children with left-sided IH, compared with 44.58% on the left in children with right-sided IH (χ^2^ = 8.869, p = 0.0029). Just 5.26% in right-sided IH vs. 4.19% in left-sided IH of adults (χ^2^ = 0.1463, p = 0.7021). The incidence of CPPV was significantly different between the right and left ipsilateral groups (left IH: χ^2^ = 58.55, p < 0.0001; right IH: χ^2^ = 114.6, p < 0.0001) (Table 2).

**Table 2:** Clinical details of unilateral IH in boys and men included in the study.

	Boys		Men		χ^2^	p
T	2386		267			
R/%	1411	59.14	191	71.54		
L/%	975	40.86	76	28.46	15.43	< 0.0001
CPPV/%	1124	47.11	12	4.49	178.1	< 0.0001
CPPV-L/%	495	50.77	4	5.26	114.6	< 0.0001
CPPV-R/%	629	44.58	8	4.19	58.55	< 0.0001
					8.869^#^	0.0029
					0.003^##^	0.9560

*CPPV-R* CPPV of right-sided IH, *CPPV-L* CPPV of left-sided IH, ^#^CPPV-R vs. CPPV-L in boys, ^##^T (theoretical frequency) = 3.42 < 5, Chi-square test with Yates' correction, CPPV-R vs. CPPV-L in adults.

### Younger vs. older children

Two groups were defined. The younger group, consisting of children under 18 months, of age, included a total of 576 cases: right-sided IH in 311 (53.99%) cases, left-sided IH in 225 (39.06%), and bilateral IH in 40 (6.94%). The mean age was 13.72 ± 3.48 months, and the mean length of medical history was 4.92 ± 4.54 months. The older group, consisting of children over 60 months, included a total of 538 cases: right-sided IH in 275 (51.12%) cases, left-sided IH in 253 (47.03%) (right-sided vs. left-sided, χ^2^ = 3.792, p = 0.0515), and bilateral IH in 10 (1.86%) (χ^2^ = 16.78, p < 0.0001). The mean age was 87.15 ± 25.30 months, and the mean length of medical history was 16.10 ± 25.71 months (t = 10.26, p < 0.0001).

A total of 256 (47.76%) CPPVs were noted in 536 unilateral IHs of the younger group, and 225 (42.61%) CPPVs were noted in 528 unilateral IHs of the older group (χ^2^ = 2.845, p = 0.0916). CPPV on the right side was identified in 48.89% of the younger group with left-sided IH, compared with 46.95% on the left in children with right-sided IH (χ^2^ = 0.1977, p = 0.6566). 49.01% in left-sided IH vs. 36.73% in right-sided IH of older group (χ^2^ = 8.132, p = 0.0043). The incidence of CPPV was significantly different on the right side between the groups (left IH: χ^2^ = 0.0007206, p = 0.9786; right IH: χ^2^ = 6.249, p = 0.0124) (Table 3).

**Table 3:** Clinical details of younger and older boys included in the study.

	Younger		Older		t/χ^2^	p
Age range (months)	≤ 18 [4–18]		≥ 60[ 60–180]			
T	576		538			
R/%	311	53.99	275	51.12		
L/%	225	39.06	253	47.03	3.792	0.0515
B/%	40	6.94	10	1.86	16.78	< 0.0001
CPPV/%	256	47.76	225	42.61	2.845	0.0916
CPPV-L/%	110	48.89	124	49.01	0.000721	0.9786
CPPV-R/%	146	46.95	101	36.73	6.249	0.0124
					0.1977^#^	0.6566
					8.132^##^	0.0043
History (months)	13.72		16.08		2.182^###^	0.0293

^#^CPPV-R vs. CPPV-L in younger children, ^##^CPPV-R vs. CPPV-L in older children, ^###^unpaired t test.

### Follow-up

**Figure 3:**
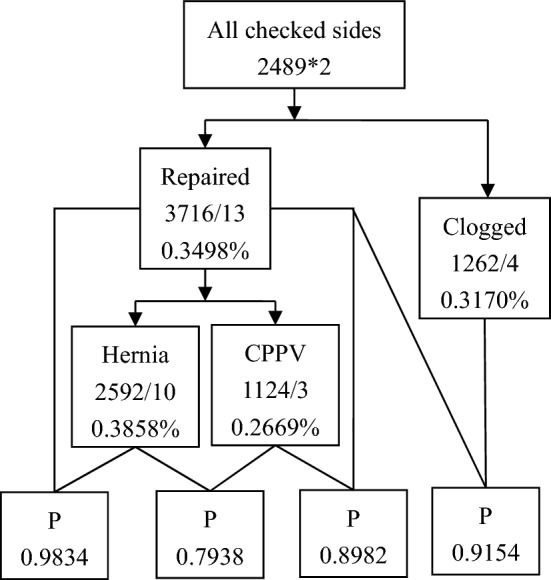
Recurrence rates by category in children.

A total of 2329 (93.57%) children were followed up for at least 2 years, and the recurrence rate of children was 0.68% (17 of 2489). The recurrence rate of contralateral clogged processus vaginalis was 0.32% (4 of 1262); the rate of recurrence on repaired sides was 0.35% (13 of 3716), consisting of 0.39% (10 of 2592) ipsilateral to repaired IHs and 0.27% (3 of 1124) ipsilateral to repaired CPPVs. There were no significant differences between these categories (Fig. 3).

## Discussion

The development of IH in boys is associated with the physiological process of testis during embryonic period^7^. The testis normally descends from its retroperitoneal location between 25 and 35 weeks of gestation, and incomplete involution results in a patent processus vaginalis (PPV)^8^. The existence of PPV is highest during infancy and declines with age^5^, as it is as high as 80% in term male infants and declines to 20–30% in adults^9^.

The younger the age group, the higher the clinical incidence of PPV is; the incidence of IH in premature infants can be as high as 30%^10^, 3%-5% in full-term infants^5^, and 0.8%-4.4% in children less than 18 years of age^11^. The data that we collected showed that 76.75% (2489/3243) of cases were children, and the age span was just 1/5 of that of adults. Although our statistics did not start from birth, both incident cases and repair procedures were most common in the early years, and the numbers of both declined rapidly, beginning at a young age (R^2^ = 0.88 and 0.97); the data imply that some cases of PPV were spontaneously obliterated with age.

The left testis descends before the testis on the right^12^, so the involution of the left PV precedes that of the right, consistent with the observation that 60% of inguinal hernias occur on the right side^5^, but IH mostly presents with bilateral IH in preterm infants^10^. The involution of bilateral PV precedes with age, and left PV obliterates first normally. Our data showed that CPPV was more common in left-sided IH than in right-sided IH.

Inguinal hernia in children is not caused by structural factors in the abdominal wall; because the PV does not close or atresia occurs during the growth process, isolated high ligation of the hernia sac can cure inguinal hernias in children^13^.

Over the past decade, laparoscopic techniques have been applied widely in the management of common pediatric diseases^14^. Laparoscopic repair in children is considered a safe, effective and convenient technique^15,16^, and CPPV can be discovered and repaired to prevent the formation of metachronous inguinal hernia (MIH)^17^. A number of studies have shown that the incidence of CPPV is 50–70%^2,3^, but the benefit from the repair is small, perhaps only 1/10^18^, 1/18^19^, or 1/21^20^, according to previous studies. However, there was no evidence that repair in CPPV can reduce the postoperative recurrence rate^21,22^. The data showed that there was no significant difference between laparoscopic repair and open repair^23^. Observation has a lower risk of morbidity than contralateral exploration^24^.

By laparoscopy, a child with a unilateral IH had more than a 50% probability of needing repair on the other side, and preventive surgery did not prevent or reduce the recurrence rate^23^. The recurrence rate of PPV is similar in repaired CPPV, contralateral clogged PV, and repaired IHs. Even so, the clogged side may develop into MIH^25^. The repairs do not decrease the risk of IH development in adulthood^26^. Moreover, the general consensus states that prevention of incarceration of IH per se is not a proper indication to perform surgery^27^, and almost all incarcerated IHs can be reduced by gentle manual pressure in children^28^. Although surgical closure of PPV is a simple procedure, significant complications remain, such as spermatic cord injury, testicular atrophy, chronic pain and infertility in adulthood^5,26,29–33^, with a tenfold increase for recurrent repair^34^. Therefore, consistent with the authors’ previous study, there was no indication for contralateral routine exploration^26,30,35–38^.

In addition, 15–37% of PPVs had no clinically apparent hernia in autopsy studies of adults, and 12% (compared to 4.49% in our study) occurred during the laparoscopic operation^16^. Compared with children, the incidence of PPV is lower. Based on the collected data, a significant number of patients' PPV does obliterate with age, and the incidence of developing hernias is similar to the incidence of hernia recurrence, so we hold the opinion that LP in CPPV results in overtreatment.

## Conclusions

IH in children is caused by the processus vaginalis not being obliterated and involuted. The processus vaginalis is formed in the embryo and closes during development but is not yet fully obliterated at birth. CPPV may develop into MIH, remain present but asymptomatic, or close over time. In the long term, most close before adulthood, a few are asymptomatic, and only a few eventually develop into MIH. Therefore, a general policy of laparoscopic IH repair in children results in overtreatment.

## Data Availability

The data that support the findings of this study are openly available in electronic medical record system of Linyi Central Hospital, reference number is 12371300495276972U.
